# Modern Sociology

**Published:** 1902-12-13

**Authors:** 


### Modern Sociology.

### THE C.E.T.S. AND WOMEN DRUNKARDS.

By a Correspondent.

Whether or not intemperance among women is on the increase, there cannot be the smallest doubt that it is an evil which may well be described as "a plague-spot," and which is a grave source of danger to future generations, not so much from the point of view of heredity, as for this reason, that drinking mothers cannot expect to have healthy children. The causes of the evil are various, and some of the chief were brought out by the speakers at a meeting at the Church House, Westminster, on Thursday, November 6th. Sir Thomas Barlow instanced: (1) lack of occupation; (2) trouble and worry; (3) bodily weakness, and the various nagging pains women have to suffer; (4) some cases of what seem to be an hereditary taste for alcohol; these, he said, were rare; (5) encouragement and example, which he believed to be much the most common cause. The Bishop of London, who followed, put his finger on two potent causes when he said, "Undoubtedly it is worry and sheer poverty, because we leave them to earn their living on wages that are inadequate. It is, as Lord Shaftesbury used to say, not so much the pig that makes the sty, as the sty that makes the pig; and the question is tremendously bound up with the better housing of the people, and with the movement to see that they have a proper wage on which they can live decently."

Among factory girls, according to a letter he had received from a lady who asked him to draw attention to this point, the existence of spirit clubs is a great cause of drinking; the girls are pressed to subscribe during the autumn, and the money is all spent on wine and spirits which are drunk on Christmas Eve, a day more to be guarded against, in the opinion of the writer, than Boxing Day. She added that these clubs were little known, and that workers among factory girls should be informed of the evil, in order that it might be combated.

In a series of articles on Shop Assistants which appeared some time ago in The Hospital, mention was made of the practice, apparently on the increase, of spirit drinking among young women employed in showrooms or behind the counter, the cause being the fatigue and exhaustion brought on by long hours and excessive standing. "The rush and pressure of life" was thought by the Bishop of Kensington to be one of the chief causes, especially in the West End.

Viscount Peel attributes the prevalence of drinking habits among women to the overwhelming number of public-houses; his argument is that the greater the temptation, the more chances there are of yielding to it. "I do not say," he says, "that if you diminish the number of public-houses you will eradicate drunkenness altogether; I will say that the more the public-houses the greater the intoxication." One of the ladies who gave evidence before the Commission of which Lord Peel was chairman, brought a very heavy charge against the grocers' shops. Many people agree with her, and even honest admirers of Mr. Gladstone consider the introduction of grocers' licenses to have been a huge blunder. This lady, Mrs. Hawkes, of Bristol, was convinced that a great quantity of so-called groceries—butter, tea, and so on—were only other names for alcohol, that alcohol was entered into the books under these harmless headings, and she was sure that this sort of thing greatly increased the consumption in the homes of the poorer classes.

What is the Church of England Temperance Society—which is able to get together a large meeting like that at the Church House—doing to arrest drunkenness among women? It has established a branch in every diocese in England and Wales, and 7,000 parochial associations, some for children, some for adults. "These parochial associations," one of the pamphlets issued by the Society states, "are doing, or ought to be doing, definite work. They should be educating public opinion; spreading the facts and principles upon which the temperance movement rests." The writer of the pamphlet plainly hopes that the parochial associations are doing this, but he does not seem quite sure that they are. And who that has ever attended temperance meetings—like the young lady of Spain, not once and again, but again, and again, and again—can fail to be struck with wonder at the zeal that makes it possible to go on holding meetings, year in and year out, in the hope of promoting temperance? The belief that public opinion is created by this means is undoubtedly what keeps up the interest. The diocesan branches would appear to exhibit greater activity, and to have a more varied programme to carry out. There are Police Court and Prison-gate Missions (and women, individually, sad to say, show a higher record of convictions than men), with over a hundred male missionaries and a dozen female (a very small number, by the way), visiting about 260 police courts in different parts of the country. Then there is an Employment Agency, for finding work for rescue cases, no easy task, judging by the Report.

Attached to these missions are, besides eight labour homes for men and boys, several shelter homes, three of which appear to be for women, while of permanent inebriates' homes there are six, three of them being Diocesan and three under the control of the central, or parent society. Over 125 women were resident during 1901; this is not the whole number, for the report of one of the homes does not give statistics, merely stating that there were 125 applications, many of which were refused, and that the Home has been a "refuge and a covert from the storm to many weak, helpless, and tempted ones." This is satisfactory as far as it goes, but we should like some more explicit information. We cannot help thinking that the society's report would be more valuable as well as interesting if the mass of material were a little more classified, and the uninitiated reader finds it somewhat difficult to gather a correct idea of where all the homes are; in one case it is only the resignation of a lady on the committee "owing to her leaving Torquay" that gives a clue to the locality of the home.

After all, statistics and information which can be tabulated do not give any idea of the individual efforts that are being made by members of this and kindred associations towards the reclamation of drunkards and the prevention which is better than cure, and it is here that the great value of such societies lies; they bring those in need of reformation into touch with the people anxious to reform them. The courageous and unremitting labour of those who meet the drunkard on the common ground of humanity and use the moral force of sympathy to win her from temptation, cannot be overrated. C.E.T.S. workers must have been greatly encouraged by the words of Sir Thomas Barlow when he advocated no half-measures in leaving off drinking habits; at least a year's residence in a home before a cure could be hoped for; and a friendly conspiracy round the patient when discharged, to see that she was employed, and that she did not contract small ailments, "and when she does," he concluded, "don't let her fall into the hands of a doctor who orders stimulants." As for the pessimistic statement that women were irreclaimable, he characterised it as "rubbish."

County Councils may in the future, even more than in the present, find they have to deal with drunkards by means of inebriates' homes, but there will always be room for the patient individual work carried on by many members of the temperance societies so long as drunkenness remains the curse it is to-day.

